# Genome-Wide Association Study and F_ST_ Analysis Reveal Four Quantitative Trait Loci and Six Candidate Genes for Meat Color in Pigs

**DOI:** 10.3389/fgene.2022.768710

**Published:** 2022-04-08

**Authors:** Hang Liu, Liming Hou, Wuduo Zhou, Binbin Wang, Pingping Han, Chen Gao, Peipei Niu, Zongping Zhang, Qiang Li, Ruihua Huang, Pinghua Li

**Affiliations:** ^1^ Institute of Swine Science, Nanjing Agricultural University, Nanjing, China; ^2^ Huaian Academy, Nanjing Agricultural University, Huaian, China; ^3^ Hangzhou Academy of Agricultural Sciences, Hangzhou, China; ^4^ Huaiyin Pig Breeding Farm of Huaian City, Huaian, China

**Keywords:** meat color, heritability, marker, GWAS, candidate genes, pigs

## Abstract

Meat color is the primary criterion by which consumers evaluate meat quality. However, there are a few candidate genes and molecular markers of meat color that were reported for pig molecular breeding. The purpose of the present study is to identify the candidate genes affecting meat color and provide the theoretical basis for meat color molecular breeding. A total of 306 Suhuai pigs were slaughtered, and meat color was evaluated at 45 min and 24 h after slaughter by CIELAB color space. All individuals were genotyped using GeneSeek GGP-Porcine 80K SNP BeadChip. The genomic estimated breeding values (GEBVs), heritability, and genetic correlation of meat color were calculated by DMU software. The genome-wide association studies (GWASs) and the fixation index (F_ST_) tests were performed to identify SNPs related to meat color, and the candidate genes within 1 Mb upstream and downstream of significant SNPs were screened by functional enrichment analysis. The heritability of L* 45 min, L* 24 h, a* 45 min, a* 24 h, b* 45 min, and b* 24 h was 0.20, 0.16, 0.30, 0.13, 0.29, and 0.22, respectively. The genetic correlation between a* (a* 45 min and a* 24 h) and L* (L* 45 min and L* 24 h) is strong, whereas the genetic correlation between b* 45 min and b* 24 h is weak. Forty-nine significant SNPs associated with meat color were identified through GWAS and F_ST_ tests. Among these SNPs, 34 SNPs were associated with L* 45 min within a 5-Mb region on Sus scrofa chromosome 11 (SSC11); 22 SNPs were associated with a* 45 min within a 14.72-Mb region on SSC16; six SNPs were associated with b* 45 min within a 4.22-Mb region on SSC13; 11 SNPs were associated with b* 24 h within a 2.12-Mb region on SSC3. These regions did not overlap with meat color–associated QTLs reported previously. Moreover, six candidate genes (*HOMER1*, *PIK3CG*, *PIK3CA*, *VCAN*, *FABP3*, and *FKBP1B*), functionally related to muscle development, phosphatidylinositol phosphorylation, and lipid binding, were detected around these significant SNPs. Taken together, our results provide a set of potential molecular markers for the genetic improvement of meat color in pigs.

## Introduction

In recent years, global meat consumption is increasing year by year (Katare et al., 2020). As an indicator of meat freshness and safety, meat color can directly affect the consumer purchase desire of pork (Tomasevic et al., 2021). The discoloration of meat surface will cause huge economic losses and is harmful to the meat industry (Suman et al., 2014). It is important for producers to use objective and scientific methods to evaluate the meat color (Wu and Sun, 2013). Currently, the CIELAB (Commission Internationale del'Éclairage LAB) color space is the most commonly used system for assessing meat color. It is a three-dimensional Cartesian space containing three mutually independent parameters, including L* (lightness), a* (redness), and b* (yellowness).

Meat color is influenced by many factors, including genetic, nutrition, and slaughter methods, among which the genetic method has a greater impact (Sellier, 1998). The heritability of meat color is low to moderate and varies among different population. Cabling et al. reported that the heritability of L*, a*, and b* was 0.44, 0.68, and 0.64 in 690 Duroc pigs, respectively (Cabling et al., 2015). However, Miar et al. reported that the heritability of meat color of 2075 offsprings from Duroc x Large White pigs was slightly lower, and the heritability of L*, a*, and b* was 0.28, 0.26, and 0.31, respectively (Miar et al., 2014). Meat quality traits have been declined because the previous swine breeding program has been focused on improving the pig’s growth rate and lean meat yield (Chen et al., 2018). However, meat quality traits are now being incorporated into the pig farm breeding objective because of the demand of the consumer market for high-quality pork (Wu et al., 2017). Traditional breeding methods are difficult to improve meat color because the determination of meat color is expensive and can only be performed after slaughter. Currently, molecular breeding technology has been widely used owing to the cost of genome sequencing, and gene chip scanning is reducing. Marker-assisted selection (MAS) is an important method of molecular breeding in which population selection is carried out through molecular markers and quantitative trait loci (QTLs) related to target traits (Borakhatariya, 2017; Visscher and Haley, 1995). The Animal QTLdb has included 651 QTLs related with meat color of pig; these QTLs are mainly distributed on the Sus scrofa chromosomes SSC6, SSC7, SSC15, and SSC16. Previous studies have reported that the *RN* gene and *PRKAG3* gene can affect the a* value of flesh color and the *RYR1* gene can improve the L* value of flesh meat (Bertram et al., 2000; Küchenmeister et al., 2000; Gunilla, 2004). Of late, the *MYH3* gene was identified associated with the a* value of meat by the genome-wide association studies (GWASs) (Cho et al., 2019).

China has more than 83 local pig breeds, and the meat quality of these local pig breeds, especially meat color, is better than Western commercial pigs, such as Landrace or Large White (Jiang et al., 2012; Lebret et al., 2015; Zhang et al., 2015). The Suhuai pig is a new cross-bred lean-type pig breed containing 25% lineage of Huai pig and 75% lineage of Large White (Wang et al., 2019). The Huai pig is one of the local pigs in North China and is well-documented for its excellent meat quality and redder meat color, while Large White is a commercial breed with a fast growth rate and poor meat quality (Yang et al., 2014; Liu et al., 2018). Briefly, after 23 years of artificial selection of the cross-bred offspring of the Large White and Huai pig, a new breed was developed, called the Xinhuai pig, which contains 50% Huai pig and 50% Large White (1954–1977). Subsequently, Large White pigs were crossed with Xinhuai pigs in 1998, and their offsprings were selected and bred for 12 years to obtain the Suhuai pig (1998–2010). The Suhuai pig is an excellent experimental population for identifying genes associated with meat color because there is phenotypic variation of meat color existent in Suhuai pig population. Moreover, the Suhuai pig’s lineage contains Huai pig lineage and Large White lineage, and the meat color of the Huai pig is better than that of Large White. These two mixed lineages may result in the differentiation in the regions of the genome that affect the Suhuai pig’s meat color. This study aims to estimate the heritability and genetic correlation of meat color and identify the candidate genes and molecular markers of meat color in Suhuai pigs, which will be beneficial for pig molecular breeding.

## Material and Methods

### Ethics Statement

All pigs were raised in accordance with the guidelines for the care and use of laboratory animals prepared by The Institute of Animal Welfare and Ethics Committee of Nanjing Agricultural University. All experimental schemes have been approved by the Animal Care and Use Committee of Nanjing Agricultural University (certificate no. SYXK (Su) 2017-0007).

### Animals and Phenotype Measurements

Three-hundred and six Suhuai pigs (227 sires and 79 dams) were used in this study. The Suhuai pigs were all fed in three batches on the Huaiyin breeding farm (Huaian, China) under the same fodder and standard management environment. The animals were slaughtered in three batches on Jinyuan Meat Products Co., Ltd. (Huaian, China). The means and standard errors of slaughter age and carcass weight were 218.3 ± 1.09 (day) and 59.1 ± 0.39 (kg), respectively. After slaughter, ear tissue samples were gathered and stored in 75% alcohol solution, and *Longissimus dorsi* (LD) muscle samples were collected from the last rib of the left half carcasses and immediately stored at 4°C. CIELAB color space of meat color was evaluated by MiniScan EZ (HunterLab Corp., New York, USA) which was calibrated according to a standard white plate. The diameter aperture was 8 mm, and D65 illuminant and 0° standard observer angle were applied. The average of the CIELAB color space from three random positions on the surface of LD muscle samples at 45 min and 24 h after slaughter (L* 45 min, L* 24 h, a* 45 min, a* 24 h, b* 45 min, and b* 24 h) was used for subsequent analyses.

### Genotyping and Quality Control

Genomic DNA was extracted from ear tissue samples following the standard phenol–chloroform method (Elder et al., 1983). All DNA samples were genotyped using the GeneSeek GGP-Porcine 80 K SNP BeadChip according to the manufacturer’s protocol. Genotype quality control was performed for selected SNPs by the PLINK 1.07 base on the follow criteria: SNP call rate ≥95%, minor allele frequency (MAF) > 1% and the *p*-value chi-square test of Hardy–Weinberg equilibrium >10^−5^ (Purcell et al., 2007). After the quality control and removing the SNPs from the sex chromosomes, 306 individuals and 52640 SNPs (Sus scrofa 11.1) were remained for subsequent analyses. The raw genotyped data of these 306 samples are available at https://doi.org/10.6084/m9.figshare.16573700.v4.

### Statistics Analyses

The mixed linear model of SAS 9.4 software (SAS Institute, Inc., Cary, NC, USA) was used to fit the fixed effects and the covariates of each CIELAB color space parameter. The relationship matrix of individuals was built based on the marker genotype information developed by VanRaden (Vanraden, 2008). The additive genetic variance and residuals of CIELAB color space parameters were calculated using AI-REML arithmetic of DMU software (Madsen, 2006), and the genomic estimated breeding values (GEBVs) and residuals of each individual were estimated using the following model:

y=μ+m+c+a+e,

where y is phenotypic observation, μ is overall mean, m is the fixed effect (L* and b* used batch and season as fixed effects; a* used batch as fixed effects), c is the covariates (L* used age and carcass weight as covariates; b* used age as covariates), a is random additive genetic effect of animal, and e is random residual error $\left[\text{e ∼ N}\left(\text{0,}\sigma_{e}^{2}\right)\right]$

The covariance between CIELAB color space parameters was calculated using the multitrait model of DMU software. The heritability and genetic correlation between CIELAB color space parameters were calculated by the following formula:

$$h^{2}=\sigma_{a}^{2}/\left(\sigma_{a}^{2}+\sigma_{e}^{2}\right),\;r_{\mathrm{gxy}}\mathrm{=co}v_{\mathrm{gxy}}/\sqrt{\sigma_{g^{x}}^{2}*\sigma_{g^{y}}^{2}},$$

where *h* ^2^ is heritability, $\sigma_{a}^{2}\;$ is additive genetic variance, $\sigma_{e}^{2}\;$ is random residual variance, r_gxy_ is the genetic correlation of trait x and y, *cov*_gxy_ is genotype covariance of trait x and y, $\sigma_{{\text{g}}^{\text{x}}}^{2}$ is additive genetic variance of trait x, and $\sigma_{g^{y}}^{2}$ is additive genetic variance of trait y.

Genome-wide association studies for meat color were performed using a single-marker regression mixed linear model of Genome-wide Efficient Mixed-Model Association (GEMMA) software (Zhou and Stephens, 2012). The model is as follows:

$$\mathrm{Y = W\alpha + x\beta + µ + \varepsilon ;µ∼MV}N_{n}\left(\mathrm{0,}\lambda_{T}^{\mathrm{-1}}k\right)\mathrm{,\varepsilon ∼ MV}N_{n}\left(0{,}_{T^{\mathrm{-1/n}}}\right),$$

where Y is the vector of the corrected phenotype that is the sum of GEBV (genomic-estimated breeding value) and residuals of individuals. W is an matrix of fixed effects that is a column of 1, *α* is a vector of the corresponding coefficient including the intercept, x is a vector of marker genotypes, *β* is the effect size of SNP, *µ* is an vector of random effects, *ε* is an vector of errors, $T^{-1}$ is the variance of the residual errors, λ is the ratio between the two variance components (genetic variance and environmental variance), K is a known relationship matrix which removed the SNPs in the same chromosome to avoid overfitting of the SNP effect on a chromosome, and MVNn denotes the dimensional multivariate normal distribution (Zhou and Stephens, 2012).

The significance threshold of the test was corrected by the Bonferroni method for GWAS; the genome-wide significance threshold was defined as 0.05/N = 8.89 * 10^−7^, and the suggestive significance threshold was defined as 1/N = 1.78 * 10^−5^ (N = the number of SNPs using in GWAS, 52640) (Yang et al., 2005).

We sorted the individuals according to the GEBV for each meat color parameter (L* 45 min, L* 24 h, a* 45 min, a* 24 h, b* 45 min, and b* 24 h), and selected the highest and lowest 30 individuals for these six parameters. GENEPOP 4.0 was used to calculate the F_ST_ statistic of each SNP for evaluating the degree of genetic differentiation in these groups (Rousset, 2008). The threshold of F_ST_ was 0.2.

### Analysis of Gene Ontology and Metabolic Pathways

The SNPs that reached both thresholds of GWAS and F_ST_ tests were used as a collective for subsequent analysis. BioMart software was used to detect candidate genes in the 1-Mb region of theses SNPs up and downstream using the Ensembl database (Hou et al., 2016). Gene Ontology (GO) term annotation and Kyoto Encyclopedia of Genes and Genomes (KEGG) analyses were performed on the annotated genes using DAVID version 6.8 (Huang et al., 2007).

## Results

### Description of Phenotypic and Genetic Parameters of Meat Color

The fixed effects and covariates of the mixed linear model for analyzing meat color were evaluated according to the significance of factors. As shown in Table 1, the batch showed an effect on L*, a*, and b*; season and age showed an effect on L* and b*; and carcass weight showed an effect on L*. Descriptive statistics and the heritability of CIELAB color space parameters are shown in Table 2. The heritability of L* 45 min, L* 24 h, a* 45 min, a* 24 h, b* 45 min, and b* 24 h was 0.20, 0.16, 0.30, 0.13, 0.29, and 0.22, respectively. The coefficient of variation of meat color ranges from 9.17% (L* 24 h) to 31.32% (a* 45 min). The genetic correlation of these parameters is shown in Table 3. Apart from b*, L* and a* showed a strong positive genetic correlation at two different time points (45 min and 24 h), which were 0.62 and 0.65, respectively. L* 45 min showed a weak negative genetic correlation with a* 45 min and a* 24 h, which are −0.45 and −0.47, respectively, but showed no genetic correlation with b*. Moreover, L* 24 h showed no genetic correlation with a* but showed genetic correlation with b* 45 min (−0.43) and b* 24 h (0.52). The genetic correlation of a* 45 min and b* were 0.62 (b* 45 min) and 0.27 (b* 24 h), respectively, and the genetic correlation of a* 24 h and b* were 0.35 (b* 45 min) and 0.70 (b* 24 h), respectively.

**TABLE 1:** Significance of the fixed effects and covariant in the mixed model for the analysis.

Parameters	N	Fixed effects			Covariant		
		Sex	Batch	Season	Age	Cw
L* 45 min	306	NS	**	*	*	*
L* 24 h	306	NS	**	**	*	*
a* 45 min	306	NS	**	NS	NS	NS
a* 24 h	306	NS	**	NS	NS	NS
b* 45 min	306	NS	*	**	**	NS
b* 24 h	306	NS	**	*	*	NS

**p < 0.05

*p < 0.01

NS, non-significant.

Cw = carcass weight.

**TABLE 2:** Descriptive statistics of meat color.

Parameters	N	Mean ± SE	Max	Min	CV (%)	*h* ^2^±SE
L* 45 min	306	40.07 ± 0.22	56.30	32.52	9.62	0.20 ± 0.10
L* 24 h	306	45.32 ± 0.24	57.40	31.04	9.17	0.16 ± 0.11
a* 45 min	306	5.14 ± 0.09	9.22	1.32	31.32	0.30 ± 0.12
a* 24 h	306	5.99 ± 0.10	14.41	2.17	28.22	0.13 ± 0.10
b* 45 min	306	11.62 ± 0.08	15.67	8.12	11.81	0.29 ± 0.11
b* 24 h	306	12.71 ± 0.09	20.45	9.22	12.33	0.22 ± 0.10

**TABLE 3:** Genetic correlation ±standard error between meat color.

Parameters	L* 45 min	L* 24 h	a* 45 min	a* 24 h	b* 45 min
L* 24 h	0.62 ± 0.04	—	—	—	—
a* 45 min	−0.45 ± 0.05	−0.14 ± 0.06	—	—	—
a* 24 h	−0.47 ± 0.05	−0.07 ± 0.06	0.65 ± 0.05	—	—
b* 45 min	−0.14 ± 0.06	−0.43 ± 0.04	0.62 ± 0.04	0.35 ± 0.05	—
b* 24 h	0.06 ± 0.06	0.52 ± 0.05	0.27 ± 0.06	0.70 ± 0.04	0.06 ± 0.07

### GWAS and F_ST_ Identified the SNPs Associated With Meat Color

**FIGURE 1:**
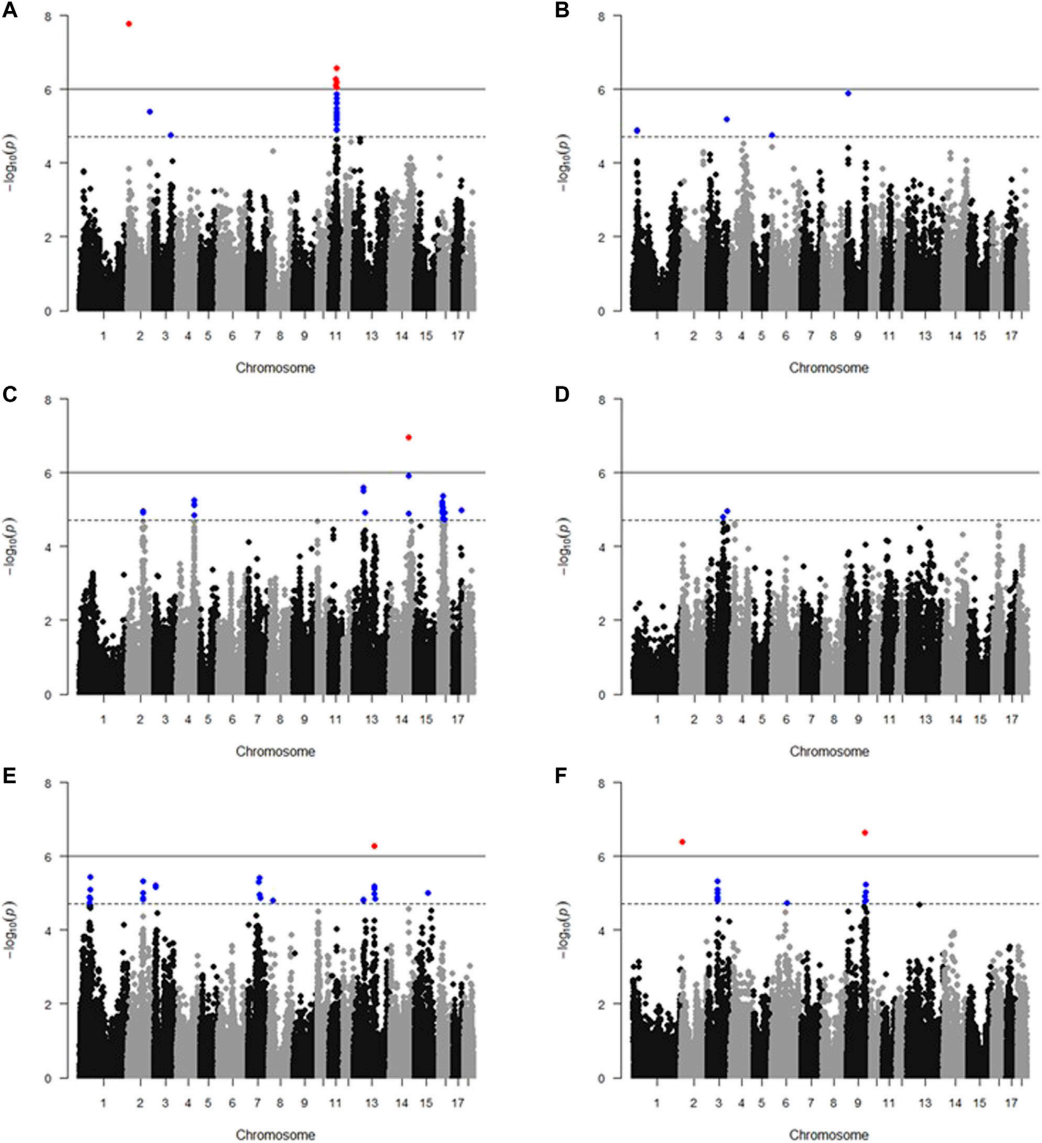
Manhattan plots of GWAS of meat color. **(A)** L* 45 min; **(B)** L* 24 h; **(C)** a* 45 min; **(D)** a* 24 h; **(E)** b* 45 min; and **(F)** b* 24 h. The *x*-axis indicates the chromosome (1-18) where the SNPs were located, and *y*-axis denotes the −log10 *p*-value. The gray dashed line represents the suggestive significance threshold (1.78 * 10^−5^), and the gray solid line represents the genome-wide significance threshold (8.89 * 10^−7^). Blue dots and red dots stand for SNPs that reached the suggestive significance threshold and genome-wide significance threshold, respectively.

The results of GWAS showed that there are 139 SNPs significantly associated with meat color, including 129 SNPs that reached the suggestive significance threshold (L* 45 min, 32 SNPs; L* 24 h, 5 SNPs; a* 45 min, 38 SNPs; a* 24 h, two SNPs; b* 45 min, 34 SNPs; and b*24 h, 18 SNPs) and 10 SNPs that reached the genome-wide significance threshold (L* 45 min, six SNPs; a* 45 min, 1 SNP; b* 45 min, one SNP; and b* 24 h, two SNPs) (Figure 1, Supplementary Table S1). It is to be noted that 34 SNPs significantly associated with L* 45 min were located in a 5.17-Mb region on SSC11 (40.13–45.30 Mb); 22 SNPs significantly associated with a* 45 min were located in a 14.72-Mb region on SSC16 (20.32–35.02 Mb); six SNPs significantly associated with b* 45 min were located in a 4.22-Mb region on SSC13 (117.69–121.91 Mb); and 11 SNPs significantly associated with b* 24 h were located in a 2.12-Mb region on SSC3 (57.52–59.64 Mb).

**FIGURE 2:**
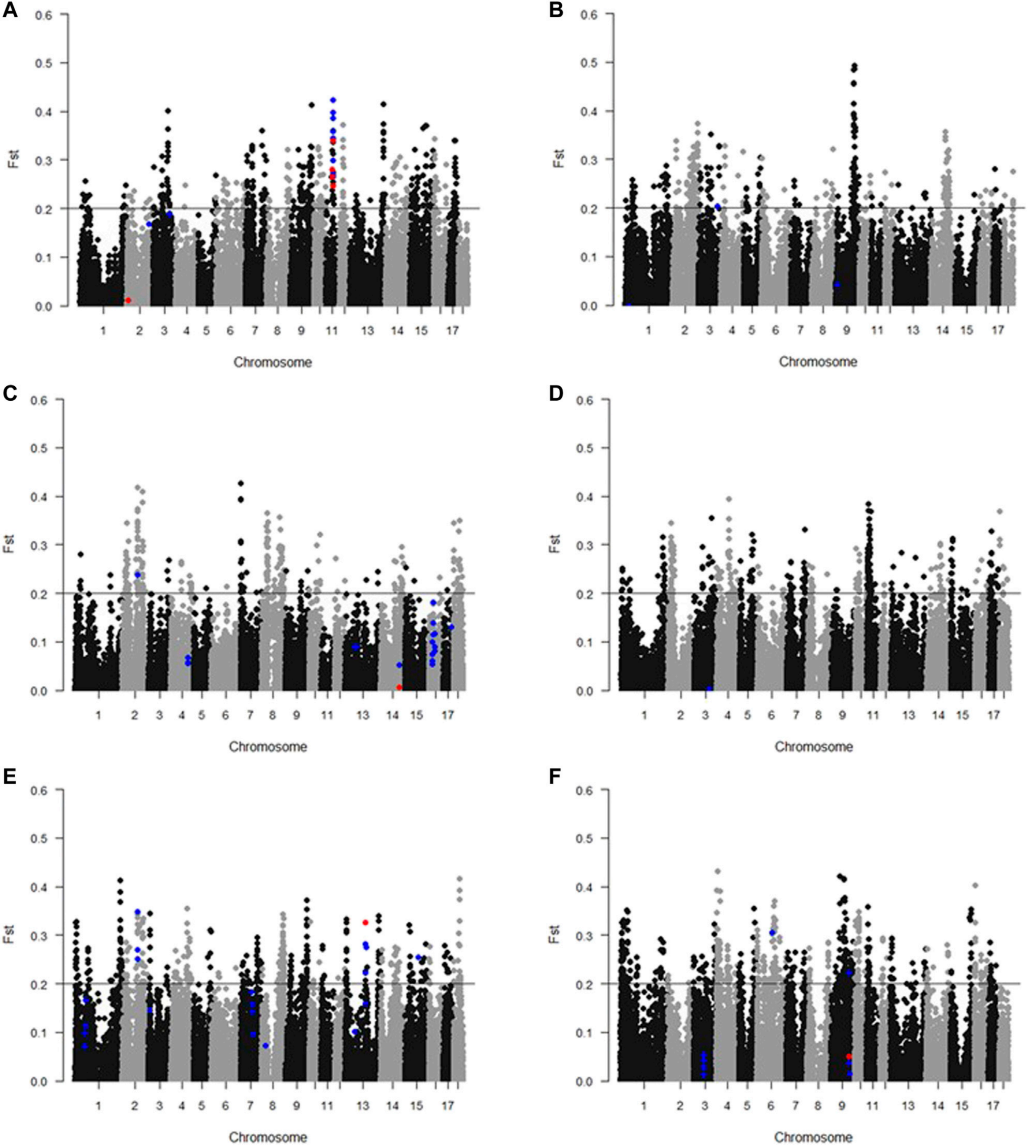
Manhattan plots of F_S_T of meat color. **(A)** L* 45 min; **(B)** L* 24 h; **(C)** a* 45 min; **(D)** a* 24 h; **(E)** b* 45 min; **(F**) b* 24 h. The *x*-axis indicates the chromosome (1-18) where the SNPs were located, and *y*-axis denotes the F_ST_ value. The line represents the threshold of differentiation (F_ST_ = 0.2). Blue dots and red dots represent SNPs that reached the suggestive significance threshold and genome-wide significance threshold, respectively.

Genome-wide fixation coefficient (F_ST_) values were calculated for each SNP between the highest and lowest individuals sorted by the GEBV for meat color. A large number of SNPs that reached the threshold (F_ST_ value >0.2) are shown in Figure 2. We focused on the overlapping results of GWAS and F_ST_ analyses. In total, 49 significant SNPs were overlapped in both GWAS and F_ST_ tests (Supplementary Table S2). Among them, 34 SNPs were identified associated with L* 45 min within a 5.17-Mb region on SSC11. Moreover, one, two, 10, and two SNPs were identified associated with L* 24 h, a* 45 min, b* 45 min, and b* 24 h, respectively.

### Identify the Candidate Genes Associated With Meat Color

**FIGURE 3:**
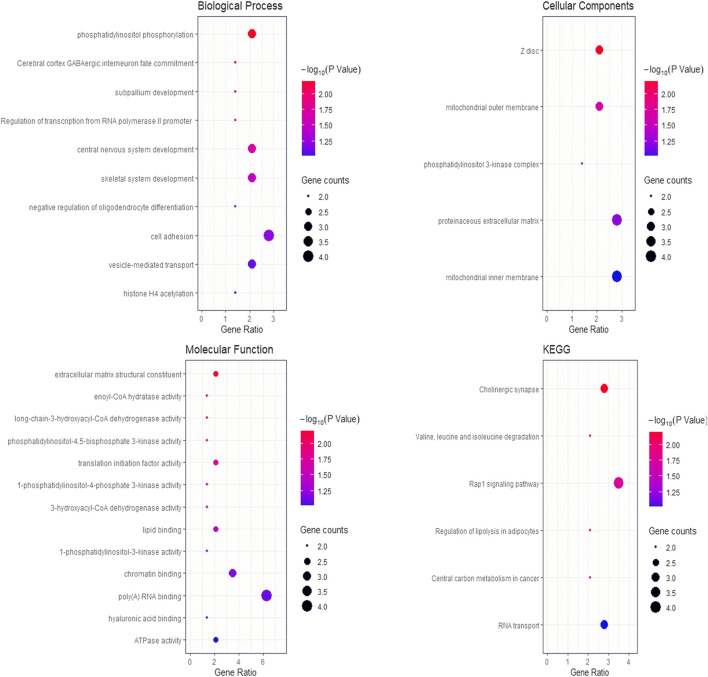
Bubble chart of GO terms and KEGG pathways for the enrichment analyses. The *y*-axis represents the gene functions or pathways and the *x*-axis is a ratio between the number of candidate genes that are annotated to the target terms to the number of background genes.

BioMart software was used to annotate the genes located within the upstream and downstream 1 Mb of significant SNPs, and 163 genes in total were identified (Supplementary Table S3). A total of 28 GO terms and six KEGG pathways were enriched by the DAVID platform (Figure 3). It is worth noting that five significant GO terms (*p* < 0.05) and one GO term which tends to be significant (*p* = 0.0501) are possibly relevant to meat color (Table 4). Six genes were identified in these terms that may affect meat color; a* 45 min (*HOMER1*), b* 45 min (*PIK3CA* and *VCAN*), b* 24 h *(FABP3* and *PIK3CG*), and L* 24 h (*FKBP1B*). These genes can be used as candidate genes of meat color in Suhuai pigs. It is noted that most of the SNPs were located in intron and intergenic regions, except rs81361290, which is located in one of the exons of a non-coding transcript (Supplementary Table S4).

**TABLE 4:** Enrichment analysis results related with meat color.

Categories	Terms	*p*-value	Genes
GOTERM_BP_DIRECT	GO:0046854∼phosphatidylinositol phosphorylation	0.0063	PIK3CG, PIK3CA, and EFR3B
GOTERM_BP_DIRECT	GO:0001501∼skeletal system development	0.0343	HAPLN1, VCAN, and CHRD
GOTERM_CC_DIRECT	GO:0030018∼Z disc	0.0463	SYNC, HOMER1, and FKBP1B
GOTERM_MF_DIRECT	GO:0046934∼phosphatidylinositol-4,5-bisphosphate 3-kinase activity	0.0268	PIK3CG and PIK3CA
GOTERM_MF_DIRECT	GO:0035005∼1-phosphatidylinositol-4-phosphate 3-kinase activity	0.0400	PIK3CG and PIK3CA
GOTERM_MF_DIRECT	GO:0008289∼lipid binding	0.0501	PFN4, FABP3, and AP2M1

## Discussion

As a direct indicator of pork quality, meat color can significantly affect the economy of the meat market. In this study, the heritability and genetic correlation of meat color were calculated by DMU software, which provided the genetic theoretical basis for molecular breeding of meat color. In order to improve the accuracy and reliability of QTLs for meat color, GWAS and F_ST_ were used in this study, and the overlapping regions identified by these two methods were used to identify candidate genes of meat color (Tang et al., 2020). The GWAS identified the candidate loci by a mixed linear model, and F_ST_ identified the candidate loci by detecting SNP differentiation between high and low groups according to GEBV. Through a combination of GWAS and F_ST_ tests, the candidate SNPs related to meat color were identified, and relevant functional candidate genes were detected by bioinformatic analysis.

The meat color at two different time points (45 min and 24 h) after slaughter was measured in this study, which represented the meat color of fresh meat and chilled meat production with different economic values. For more effectively and accurately measuring meat color, the CIELAB color space was used. In this study, the effects affecting meat color are different, but the batch showed significant effect on meat color that may be due to the difference of the environment. The season showed significant effect on L* and b* but has no significant effect on a*. a* is mainly related to the content and state of myoglobin, while L* and b* are greatly affected by the biochemical reaction of muscles which may be affected by temperature and humidity. It was reported that a* was related to the proportion of muscle fiber types in the skeletal muscle (Kim, 2010). The value of a* was relatively high when the skeletal muscle is dominated by slow-oxidative muscle fibers, which have a high content of myoglobin (Vierck et al., 2018). L* was identified not only related to the proportion of fiber types in the muscle, but it is also related to the glycogen content and the ability of glycolysis in the muscle (Ryu et al., 2008). Meanwhile, L*, especially L* 24 h, was affected by the fiber structure in the muscle, which determines the light absorption and reflection ability of the meat (Hughes et al., 2020).Studies have reported that b* could be affected by lipid (Ha et al., 2017). Meat color was affected by factors which were influenced by the storage environment; therefore, it can be seen that the heritability of meat color at 24 h was less than that at 45 min. The heritability of meat color ranges from 0.1 to 0.3, which belongs to low and middle heritability, and this results are consistent with other reports (Khanal et al., 2019).

We defined |R|< 0.2 as irrelevant, 0.2 < |R| < 0.5 as weak correlation, and |R| > 0.5 as strong correlation. Our results showed that the genetic correlation of a* (a* 45 min and a* 24 h) and L* (L* 45 min and L* 24 h) is strong, but b* 45 min and b* 24 h have no genetic correlation with each other, indicating that the genetic background of b*45 min and b* 24 h may be different. Our results showed that the main influencing factors for a* 45 min and a* 24 h are similar, whereas the main influencing factors of b* 45 min and b* 24 h could be different. There was a weak negative genetic correlation between L* 45 min and a* (a* 45 min and a* 24 h), which may be related to the proportion of muscle fiber types (Hughes et al., 2020). There is no genetic correlation between L* 24 h and a* (a* 45 min and a* 24 h), indicating that L* 24 h may be more affected by other factors such as pH, water-holding capacity, and structure of muscle fibers etc. It is worth noting that L* 24 h has a weak negative genetic correlation with b* 45 min and strong positive genetic correlation with b* 24 h, which indicated that the main influencing factors of b* 45 min and b* 24 h are different. The a* and b* showed strong positive genetic correlation at the same time point and weak positive genetic correlation at different time points, indicating that although b* is complex, it may have the same genetic background with a*. Indeed, it is noteworthy that the parameter of meat color could affect each other.

Among the meat color of Suhuai pigs, the variation coefficient of a* is the largest, which is over 30%, while the variation coefficient of L* is over 9%. Therefore, SNPs and genes affecting meat color could be identified by GWAS in Suhuai pigs. In order to reduce the false-positive rate and improve the power of the GWAS model and F_ST_ tests, we used GEBV plus residual as the corrected phenotype. In total, we identified 49 SNPs and both reached the significance threshold of F_ST_ value and GWAS, which could act as the candidate sites associated with meat color in this study. Interestingly, the parameter at the two time points after slaughter did not share the same significant SNPs, which indicated that the main influencing factors of meat color at 45 min and 24 h after slaughter may be different from a genetic perspective. The meat color at 24 h after slaughter may be mainly affected by metabolic reactions in the muscle, such as glycolysis reaction of the muscle after slaughter; however, the meat color at 45 min after slaughter may be primarily determined by the content of muscle substances such as myoglobin, fat, and moisture etc. These SNPs were not overlapped with the previously reported QTL intervals related with meat color. Meat color is a complex economic trait which is regulated by complex genetic networks, and the genes causing the different meat color in different pig breeds may be located at different regulatory network nodes, which may be the reasons why the current study identified a few new associated genetic regions that were not identified by previous studies.

Although meat color was evaluated using different parameters (L*, a*, and b*) at different time points (45 min and 24 h) after slaughter, the genetic correlation of meat color parameters range from −0.47 to 0.70 (Table 3). Therefore, genes within 1 Mb upstream and downstream of all significant sites were used as a collective for functional enrichment analyses. The results enriched multiple pathways, including muscle development (GO:0001501 and GO:0030018), phosphatidylinositol phosphorylation (GO:0046854, GO:0046934, and GO:0035005) and lipid binding (GO:0008289). Phosphatidylinositol is involved in a variety of physiological functions in the body, including muscle contraction, cell proliferation, and differentiation. The genes within the region on SSC11 (40.13–45.30 Mb) related to L* 45 min were not enriched in any pathway and were not reported to affect meat color. It is possible that there is a regulatory element in this region that regulates the expression of downstream genes. In total, we identified six candidate genes in these pathways related to meat color. Of these candidate genes, only the *HOMER1* gene was associated with muscle development, and the rs81360833 (*p* = 1.21E-05) was suggestive to be significantly associated with a* 45 min and was located in the region of the *HOMER1* gene. Homer1 is one of the homer family members that play a role in activity-dependent control of neuronal responses (Worley, 1998). As the scaffolding protein, the lack of Homer1 can cause the dysregulation of transient receptor potential (TRP) channels. It was reported that mice lacking Homer1 showed the decreasing of the muscle fiber cross-sectional area and skeletal muscle force generation, which may cause increasing spontaneous calcium influx (Michel et al., 2004; Stiber et al., 2008). The *HOMER1* gene has different expression patterns in the skeletal muscle of three different pig breeds, including Large White (lean-type), Tongcheng (obese-type), and Wuzhishan (mini-type) (Hou et al., 2016). These studies suggested that *HOMER1* may play an important regulatory role during skeletal muscle growth, which could affect the proportion of muscle fiber types in the skeletal muscle and resulted in different redness (a*) of the skeletal muscle.

Four candidate genes associated with the b* were identified, which were involved in the physiological function of fat deposition. The *PIK3CA* gene encoded the P110α protein, which is a member of the enzyme phosphoinositide 3-kinase (PI3k) family and plays an important role in glucose metabolism, angiogenesis, and cellular growth. *PIK3CA* is a key mediator in insulin signaling, which can regulate glucose and lipid metabolism and the expression of major gluconeogenic-related genes (Sopasakis et al., 2010). The *PIK3CA* gene was differentially expressed in the two groups which were divided according to the degree of fat deposition in the muscle and enriched in the pathways related to the differentiation of adipose tissue (Cánovas et al., 2010). P110γ, encoded by the *PIK3CG* gene, is the unique catalytic subunit of the PI3K family, and it is involved in the Akt pathway of glucose transport and fat production (Puig-Oliveras et al., 2014). Studies related to the *PIK3CG* gene were mainly focused on signal transduction of inflammation, and p110γ is a major driver of metabolic diseases, such as fatty liver disease and type-2 diabetes (Van Greevenbroek et al., 2013). The *PIK3CG* gene has been identified as a candidate gene affecting intramuscular fat (IMF) and fatty acid (FA) in the swine muscle of Iberian X Landrace backcross animals (Puig-Oliveras et al., 2014). Versican (VCAN), is considered critical to several key cellular processes which may influenced the growth of adipose tissue, including cellular adhesion, proliferation, differentiation, migration, and angiogenesis (Du et al., 2011). It has been reported that the *VCAN* gene is associated with glucose tolerance in obese patients (Minchenko et al., 2013). The *VCAN* gene is associated with pork quality and fat deposition in pork (Piorkowska et al., 2018). Cardiac fatty acid–binding proteins (FABP3) participate in lipid metabolism by ingesting or utilizing long-chain fatty acids. An SNP located in *FABP3* promoter region was found in purebred Large White, Duroc, and Pietrain populations, which was identified related to adipogenesis (Sweeney et al., 2015). These four candidate genes (*PIK3CA*, *PIK3CG*, *VCAN*, and *FABP3*) have been reported to be involved in the regulation of fat metabolism pathways and affected the changes of fatty acid content and glycogen content in the muscle, which could be one of the reasons for the variation of yellowness (b*).

Genes located in the region of L* 45 min–associated SNPs were not enriched into any pathways; thus, further studies are needed to reveal the genetic basis for L* 45 min in other pig breeds. The *FKBP1B* gene was identified near the significant SNP of L* 24 h. FKBP1B is a member of the peptide-proline isomerase family and can be detected in a variety of cells. Studies have found that mir-34a mimic can regulate fat production by reducing the expression of *FKBP1B* mRNA in preadipocytes, indicating the importance of FKBP1B in fat production (Jang et al., 2015). In addition to muscle fiber types and the structure of the muscle fiber, L* may be affected by *FKBP1B* through fat metabolism.

## Conclusions

The a* value of meat color has a large degree of variation in Suhuai pigs. The heritability of L* 45 min, L* 24 h, a* 45 min, a* 24 h, b* 45 min, and b* 24 h was 0.20, 0.16, 0.30, 0.13, 0.29, and 0.22, respectively. The genetic correlation between a* (a* 45 min and a* 24 h) and L* (L* 45 min and L*24 h) is strong. Forty-nine potential meat color–related SNPs were identified using GWAS and F_ST_ tests in Suhuai pigs, and six candidate genes (*HOMER1*, *PIK3CG*, *PIK3CA*, *VCAN*, *FABP3*, and *FKBP1B*), which are functionally related to muscle development, phosphatidylinositol phosphorylation, and lipid binding, were detected around these significant SNPs. These findings provide theoretical and molecular basis for genetic improvement of meat color in pigs.

## Data Availability

The original contributions presented in the study are included in the article/Supplementary Material, further inquiries can be directed to the corresponding author.

## Supplementary Material

Supplementary Figure S1 PCA analysis of experimental population.

## References

[B1] Bertram H. C. Petersen J. S. Andersen H. J. (2000). Relationship between RN− Genotype and Drip Loss in Meat from Danish Pigs. Meat Sci. 56 (1), 49–55. 10.1016/S0309-1740(00)00018-8 22061770

[B2] Borakhatariya D. (2017). Genomic Selection in Dairy Cattles: a Review. Int. J. Sci. Environ. Technology 6 (1), 339–347. 10.20546/ijcmas.2017.608.069

[B3] Cabling M. M. Kang H. S. Lopez B. M. Jang M. Kim H. S. Nam K. C. (2015). Estimation of Genetic Associations between Production and Meat Quality Traits in Duroc Pigs. Asian Australas. J. Anim. Sci. 28, 1061–1065. 10.5713/ajas.14.0783 26104512 PMC4478472

[B4] Cánovas A. Quintanilla R. Amills M. Pena R. N. (2010). Muscle Transcriptomic Profiles in Pigs with Divergent Phenotypes for Fatness Traits. BMC Genomics 11 (372). 10.1186/1471-2164-11-372 PMC2894043 20540717

[B5] Cheng J. Newcom D. W. Schutz M. M. Cui Q. Li B. Zhang H. (2018). Evaluation of Current United States Swine Selection Indexes and Indexes Designed for Chinese Pork Production. The Prof. Anim. Scientist 34 (5), 474–487. 10.15232/pas.2018-01731

[B6] Cho I.-C. Park H.-B. Ahn J. S. Han S.-H. Lee J.-B. Lim H.-T. (2019). A Functional Regulatory Variant of MYH3 Influences Muscle Fiber-type Composition and Intramuscular Fat Content in Pigs. Plos Genet. 15 (10), e1008279. 10.1371/journal.pgen.1008279 31603892 PMC6788688

[B7] Du W. W. Yang B. B. Yang B. L. Deng Z. Fang L. Shan S. W. (2011). Versican G3 Domain Modulates Breast Cancer Cell Apoptosis: a Mechanism for Breast Cancer Cell Response to Chemotherapy and EGFR Therapy. PLoS One 6 (11), e26396. 10.1371/journal.pone.0026396 22096483 PMC3212514

[B8] Elder R. T. Fritsch F. E. Maniatis T. (1983). Cloning Techniques Molecular Cloning: A Laboratory Manual T. Maniatis E. R. Pritsch J. Sambrook. BioScience 33, 721–722. 10.2307/1309366

[B9] Gunilla L. (2004). A Second Mutant Allele (V199I) at the PRKAG3 (RN) Locus-II. Effect on Colour Characteristics of Pork Loin. Meat Sci. 66 (3), 621–627. 10.1016/S0309-1740(03)00180-3 22060872

[B10] Ha J. Kwon S. Hwang J. H. Park D. H. Kim T. W. Kang D. G. (2017). Squalene Epoxidase Plays a Critical Role in Determining Pig Meat Quality by Regulating Adipogenesis, Myogenesis, and ROS Scavengers. Sci. Rep. 7 (1), 16740–16749. 10.1038/s41598-017-16979-x 29196684 PMC5711910

[B11] Hou X. Yang Y. Zhu S. Hua C. Zhou R. Mu Y. (2016). Comparison of Skeletal Muscle miRNA and mRNA Profiles Among Three Pig Breeds. Mol. Genet. Genomics 291, 559–573. 10.1007/s00438-015-1126-3 26458558

[B12] Huang D. W. Sherman B. T. Tan Q. Kir J. Liu D. Bryant D. (2007). DAVID Bioinformatics Resources: Expanded Annotation Database and Novel Algorithms to Better Extract Biology from Large Gene Lists. Nucleic Acids Res. 35 (2), W169–W175. 10.1093/nar/gkm415 17576678 PMC1933169

[B13] Hughes J. M. Clarke F. M. Purslow P. P. Warner R. D. (2020). Meat Color Is Determined Not Only by Chromatic Heme Pigments but Also by the Physical Structure and Achromatic Light Scattering Properties of the Muscle. Compr. Rev. Food Sci. Food Saf. 19 (1), 44–63. 10.1111/1541-4337.12509 33319522

[B14] Jang Y. J. Jung C. H. Ahn J. Gwon S. Y. Ha T. Y. (2015). Shikonin Inhibits Adipogenic Differentiation *via* Regulation of Mir-34a-Fkbp1b. Biochem. Biophysical Res. Commun. 467, 941–947. 10.1016/j.bbrc.2015.10.039 26471303

[B15] Jiang Y. Z. Zhu L. Tang G. Q. Li M. Z. Jiang A. A. Cen W. M. (2012). Carcass and Meat Quality Traits of Four Commercial Pig Crossbreeds in China. Genet. Mol. Res. 11 (44), 4447–4455. 10.4238/2012.September.19.6 23079983

[B49] Kim G. D. Jeong J. Y. Hur S. J. Yang H. S. Joo S. T. (2010). The Relationship between Meat Color (CIE L^*^ and a^*^), Myoglobin Content, and Their Influence on Muscle Fiber Characteristics and Pork Quality. Korean Journal for Food Science of Animal Resources 30 (4), 626–633. 10.5851/kosfa.2010.30.4.626

[B16] Katare B. Wang H. H. Lawing J. Hao N. Park T. Wetzstein M. (2020). Toward Optimal Meat Consumption. Am. J. Agric. Econ. 102 (2), 662–680. 10.1002/ajae.12016

[B17] Khanal P. Maltecca C. Schwab C. Gray K. Tiezzi F. (2019). Genetic Parameters of Meat Quality, Carcass Composition, and Growth Traits in Commercial Swine. J. Anim. Sci. 97 (9), 3669–3683. 10.1093/jas/skz247 31350997 PMC6735811

[B18] Küchenmeister U. Kuhn G. Ender K. (2000). Seasonal Effects on Ca2+ Transport of Sarcoplasmic Reticulum and on Meat Quality of Pigs with Different Malignant Hyperthermia Status. Meat Sci. 55, 239–245. 10.1016/S0309-1740(99)00149-7 22061090

[B19] Lebret B. Ecolan P. Bonhomme N. Méteau K. Prunier A. (2015). Influence of Production System in Local and Conventional Pig Breeds on Stress Indicators at slaughter, Muscle and Meat Traits and Pork Eating Quality. Animal 9 (8), 1404–1413. 10.1017/S1751731115000609 25908582

[B20] Liu Y. Yang X. Jing X. He X. Wang L. Liu Y. (2018). Transcriptomics Analysis on Excellent Meat Quality Traits of Skeletal Muscles of the Chinese Indigenous Min Pig Compared with the Large white Breed. Ijms 19 (1), 21. 10.3390/ijms19010021 PMC5795972 29271915

[B21] Madsen P. (2006). “DMU - A Package for Analyzing Multivariate Mixed Models in Quantitative Genetics and Genomics,” in World Congress on Genetics Applied to Livestock Production.

[B22] Miar Y. Plastow G. S. Moore S. S. Manafiazar G. Charagu P. Kemp R. A. (2014). Genetic and Phenotypic Parameters for Carcass and Meat Quality Traits in Commercial Crossbred Pigs1. J. Anim. Sci. 92 (7), 2869–2884. 10.2527/jas.2014-7685 24778330

[B23] Michel R. N. Dunn S. E. Chin E. R. (2004). Calcineurin and Skeletal Muscle Growth. Proc. Nutr. Soc. 63 (2), 341–349. 10.1079/PNS2004362 15294053

[B24] Minchenko D. Ratushna O. Bashta Y. Herasymenko R. Minchenko O. (2013). The Expression of TIMP1, TIMP2, VCAN, SPARC, CLEC3B and E2F1 in Subcutaneous Adipose Tissue of Obese Males and Glucose Intolerance. CellBio 02 (02), 45–53. 10.4236/cellbio.2013.22006

[B25] Piórkowska K. Żukowski K. Ropka-Molik K. Tyra M. Gurgul A. (2018). A Comprehensive Transcriptome Analysis of Skeletal Muscles in Two Polish Pig Breeds Differing in Fat and Meat Quality Traits. Genet. Mol. Biol. 41 (1), 125–136. 10.1590/1678-4685-gmb-2016-0101 29658965 PMC5901489

[B26] Puig-Oliveras A. Ramayo-Caldas Y. Corominas J. Estellé J. Pérez-Montarelo D. Hudson N. J. (2014). Differences in Muscle Transcriptome Among Pigs Phenotypically Extreme for Fatty Acid Composition. PLoS One 9 (6), e99720. 10.1371/journal.pone.0099720 24926690 PMC4057286

[B27] Purcell S. Neale B. Todd-Brown K. Thomas L. Ferreira M. A. R. Bender D. (2007). PLINK: a Tool Set for Whole-Genome Association and Population-Based Linkage Analyses. Am. J. Hum. Genet. 81 (3), 559–575. 10.1086/519795 17701901 PMC1950838

[B28] Rousset F. (2008). genepop'007: a Complete Re-implementation of the Genepop Software for Windows and Linux. Mol. Ecol. Resour. 8 (1), 103–106. 10.1111/j.1471-8286.2007.01931.x 21585727

[B29] Ryu Y. C. Choi Y. M. Lee S. H. Shin H. G. Choe J. H. Kim J. M. (2008). Comparing the Histochemical Characteristics and Meat Quality Traits of Different Pig Breeds. Meat Sci. 80 (2), 363–369. 10.1016/j.meatsci.2007.12.020 22063341

[B30] Sellier P. (1998). Genetics of Meat and Carcass Traits. The Genet. pig, 463–510.

[B31] Sopasakis V. R. Liu P. Suzuki R. Kondo T. Winnay J. Tran T. T. (2010). Specific Roles of the P110α Isoform of Phosphatidylinsositol 3-Kinase in Hepatic Insulin Signaling and Metabolic Regulation. Cel Metab. 11 (3), 220–230. 10.1016/j.cmet.2010.02.002 PMC3144706 20197055

[B32] Stiber J. A. Zhang Z.-S. Burch J. Eu J. P. Zhang S. Truskey G. A. (2008). Mice Lacking Homer 1 Exhibit a Skeletal Myopathy Characterized by Abnormal Transient Receptor Potential Channel Activity. Mol. Cel Biol 28 (8), 2637–2647. 10.1128/MCB.01601-07 PMC2293116 18268005

[B33] Suman S. P. Hunt M. C. Nair M. N. Rentfrow G. (2014). Improving Beef Color Stability: Practical Strategies and Underlying Mechanisms. Meat Sci. 98 (3), 490–504. 10.1016/j.meatsci.2014.06.032 25041654

[B34] Sweeney T. O'halloran A. M. Hamill R. M. Davey G. C. Gil M. Southwood O. I. (2015). Novel Variation in the FABP3 Promoter and its Association with Fatness Traits in Pigs. Meat Sci. 100, 32–40. 10.1016/j.meatsci.2014.09.014 25306509

[B35] Tang Z. Fu Y. Xu J. Zhu M. Li X. Yu M. (2020). Discovery of Selection‐driven Genetic Differences of Duroc, Landrace, and Yorkshire Pig Breeds by EigenGWAS and F St Analyses. Anim. Genet. 51 (4), 531–540. 10.1111/age.12946 32400898

[B36] Tomasevic I. Djekic I. Font-i-Furnols M. Terjung N. Lorenzo J. M. (2021). Recent Advances in Meat Color Research. Curr. Opin. Food Sci. 41, 81–87. 10.1016/j.cofs.2021.02.012

[B37] Van Greevenbroek M. M. Schalkwijk C. G. Stehouwer C. D. (2013). Obesity-associated Low-Grade Inflammation in Type 2 Diabetes Mellitus: Causes and Consequences. Neth. J. Med. 71, 174–187. 10.1007/s11845-013-0958-2 23723111

[B38] Vanraden P. M. (2008). Efficient Methods to Compute Genomic Predictions. J. Dairy Sci. 91 (11), 4414–4423. 10.3168/jds.2007-0980 18946147

[B39] Vierck K. R. O’Quinn T. G. Noel J. A. Houser T. A. Boyle E. A. E. Gonzalez J. M. (2018). Effects of Marbling Texture on Muscle Fiber and Collagen Characteristics. Meat Muscle Biol. 2 (1), 75–83. 10.22175/mmb2017.10.0054

[B40] Visscher P. Haley C. (1995). Utilizing Genetic Markers in Pig Breeding Programmes. Anim. Breed. Abstr. 63 (1), 1–8.

[B41] Wang B. Li P. Zhou W. Gao C. Liu H. Li H. (2019). Association of Twelve Candidate Gene Polymorphisms with the Intramuscular Fat Content and Average Backfat Thickness of Chinese Suhuai Pigs. Animals 9 (11), 858. 10.3390/ani9110858 PMC6912197 31652864

[B42] Worley P. F. (1998). Homer Regulates the Association of Group 1 Metabotropic Glutamate Receptors with Multivalent Complexes of homer-related, Synaptic Proteins. Neuron 21 (4), 707–716. 10.1016/S0896-6273(00)80588-7 9808458

[B43] Wu D. Sun D.-W. (2013). Colour Measurements by Computer Vision for Food Quality Control - A Review. Trends Food Sci. Technology 29 (1), 5–20. 10.1016/j.tifs.2012.08.004

[B44] Wu F. Vierck K. R. Derouchey J. M. O'Quinn T. G. Tokach M. D. Goodband R. D. (2017). A Review of Heavy Weight Market Pigs: Status of Knowledge and Future Needs Assessment1. Anim. Sci. 1 (1), 1–15. 10.2527/tas2016.0004 PMC7235466 32704624

[B45] Yang J. Zhou L. Liu X. Ma H. Xie X. Xiong X. (2014). A Comparative Study of Meat Quality Traits between Laiwu and DLY Pigs. Acta Veterinaria et Zootechnica Sinica 45, 1752–1759.

[B46] Yang Q. Cui J. Chazaro I. Cupples L. A. Demissie S. (2005). Power and Type I Error Rate of False Discovery Rate Approaches in Genome-wide Association Studies. BMC Genet. 6, 134–137. 10.1186/1471-2156-6-S1-S134 PMC1866802 16451593

[B47] Zhang C. Luo J. Q. Zheng P. Yu B. Huang Z. Q. Mao X. B. (2015). Differential Expression of Lipid Metabolism-Related Genes and Myosin Heavy Chain Isoform Genes in Pig Muscle Tissue Leading to Different Meat Quality. Animal 9 (06), 1073–1080. 10.1017/S1751731115000324 25716066

[B48] Zhou X. Stephens M. (2012). Genome-wide Efficient Mixed-Model Analysis for Association Studies. Nat. Genet. 44 (7), 821–824. 10.1038/ng.2310 22706312 PMC3386377

