# Supplementary material for: Genome-Wide Association Study and FST Analysis Reveal Four Quantitative Trait Loci and Six Candidate Genes for Meat Color in Pigs
Source: Front Genet. 2022 Apr 8;13:768710. doi: 10.3389/fgene.2022.768710 (PMC9023761; doi:10.3389/fgene.2022.768710)
Supplement: Supplementary file 1 [file DataSheet1.docx]

**Table S1** Significant SNPs associated with meat color were identified by GWAS

| Parameters | | CHR | SNP | BP | P-value |
| --- | --- | --- | --- | --- | --- |
| L* 45min | | 2 | WU_10.2_2_8181349 | 8838326 | 1.7E-08 |
|  |  | 2 | WU_10.2_2_134560513 | 129271824 | 4.01E-06 |
|  |  | 3 | ALGA0020271 | 97178250 | 1.74E-05 |
|  |  | 3 | DIAS0002455 | 97201560 | 1.74E-05 |
|  |  | 11 | WU_10.2_11_49017756 | 44556329 | 2.71E-07 |
|  |  | 11 | DRGA0011179 | 40134152 | 5.24E-07 |
|  |  | 11 | WU_10.2_11_48411413 | 44005756 | 6.48E-07 |
|  |  | 11 | WU_10.2_11_44425665 | 40691397 | 7.97E-07 |
|  |  | 11 | WU_10.2_11_46911585 | 42772353 | 8.8E-07 |
|  |  | 11 | ALGA0123552 | 43676387 | 1.32E-06 |
|  |  | 11 | H3GA0031860 | 41476323 | 1.76E-06 |
|  |  | 11 | DRGA0011191 | 41250991 | 2.3E-06 |
|  |  | 11 | ASGA0094138 | 40874619 | 2.36E-06 |
|  |  | 11 | DRGA0011182 | 40995311 | 2.36E-06 |
|  |  | 11 | H3GA0031845 | 41014426 | 2.36E-06 |
|  |  | 11 | ASGA0050710 | 41097675 | 2.36E-06 |
|  |  | 11 | ASGA0050717 | 41328254 | 2.36E-06 |
|  |  | 11 | DRGA0011196 | 41361792 | 2.36E-06 |
|  |  | 11 | ASGA0091324 | 42337689 | 3.25E-06 |
|  |  | 11 | WU_10.2_11_47243159 | 42922606 | 4.02E-06 |
|  |  | 11 | MARC0105378 | 41831777 | 4.47E-06 |
|  |  | 11 | DRGA0011209 | 41884238 | 4.47E-06 |
|  |  | 11 | ALGA0062058 | 41962603 | 4.47E-06 |
|  |  | 11 | ALGA0062070 | 42016587 | 4.47E-06 |
|  |  | 11 | DRGA0011217 | 42054759 | 4.47E-06 |
|  |  | 11 | ALGA0062067 | 42138666 | 4.47E-06 |
|  |  | 11 | MARC0114526 | 42196800 | 4.47E-06 |
|  |  | 11 | MARC0042558 | 42215359 | 4.47E-06 |
|  |  | 11 | ALGA0062020 | 41123461 | 4.93E-06 |
|  |  | 11 | WU_10.2_11_46097748 | 41821272 | 5.44E-06 |
|  |  | 11 | DRGA0011202 | 41458769 | 5.87E-06 |
|  |  | 11 | DRGA0011201 | 41441041 | 6.38E-06 |
|  |  | 11 | WU_10.2_11_49737400 | 45309679 | 6.92E-06 |
|  |  | 11 | ALGA0062080 | 42398152 | 9.03E-06 |
|  |  | 11 | ALGA0062050 | 41772581 | 1.24E-05 |
|  |  | 11 | H3GA0031866 | 42151978 | 1.24E-05 |
|  |  | 11 | WU_10.2_11_46431693 | 42187347 | 1.24E-05 |
|  |  | 11 | MARC0069213 | 44662760 | 1.29E-05 |
| L* 24h | | 1 | INRA0001175 | 24260803 | 1.3E-05 |
|  |  | 1 | H3GA0001142 | 24114687 | 1.36E-05 |
|  |  | 3 | WU_10.2_3_120769648 | 113665225 | 6.55E-06 |
|  |  | 6 | ALGA0103513 | 3789349 | 1.72E-05 |
|  |  | 9 | H3GA0026345 | 8891700 | 1.27E-06 |
| a* 45min | | 2 | ASGA0010795 | 88083522 | 1.09E-05 |
|  |  | 2 | ALGA0014301 | 88166097 | 1.21E-05 |
|  |  | 4 | H3GA0013765 | 100012642 | 5.64E-06 |
|  |  | 4 | ASGA0021380 | 99817682 | 7.19E-06 |
|  |  | 4 | ASGA0021392 | 99967847 | 7.73E-06 |
|  |  | 4 | WU_10.2_4_109273333 | 99670128 | 1.39E-05 |
|  |  | 13 | ASGA0094034 | 56653519 | 2.53E-06 |
|  |  | 13 | CASI0004415 | 57384026 | 3.16E-06 |
|  |  | 13 | ASGA0057943 | 64696229 | 1.21E-05 |
|  |  | 13 | ALGA0107703 | 64729255 | 1.21E-05 |
|  |  | 13 | ASGA0057953 | 64789716 | 1.21E-05 |
|  |  | 14 | ALGA0038327 | 109305728 | 1.07E-07 |
|  |  | 14 | H3GA0042042 | 110891845 | 1.23E-06 |
|  |  | 14 | ALGA0080999 | 109616495 | 1.29E-05 |
|  |  | 16 | ALGA0123380 | 26362525 | 4.33E-06 |
|  |  | 16 | MARC0097177 | 20491365 | 6.2E-06 |
|  |  | 16 | MARC0086770 | 20525463 | 6.2E-06 |
|  |  | 16 | WU_10.2_16_21444165 | 20542197 | 6.2E-06 |
|  |  | 16 | MARC0083773 | 20559236 | 6.2E-06 |
|  |  | 16 | H3GA0046184 | 20326495 | 7.2E-06 |
|  |  | 16 | WU_10.2_16_21317885 | 20416579 | 7.8E-06 |
|  |  | 16 | ASGA0072531 | 20429883 | 7.8E-06 |
|  |  | 16 | WU_10.2_16_25424322 | 24177060 | 8.85E-06 |
|  |  | 16 | WU_10.2_16_22185838 | 21165261 | 1.11E-05 |
|  |  | 16 | ASGA0072792 | 25918846 | 1.19E-05 |
|  |  | 16 | ALGA0090278 | 35019084 | 1.2E-05 |
|  |  | 16 | MARC0007989 | 20891745 | 1.21E-05 |
|  |  | 16 | WU_10.2_16_21877990 | 20909358 | 1.21E-05 |
|  |  | 16 | DIAS0002401 | 32143470 | 1.23E-05 |
|  |  | 16 | ASGA0072801 | 25956414 | 1.69E-05 |
|  |  | 16 | DIAS0003044 | 26030170 | 1.69E-05 |
|  |  | 16 | H3GA0046313 | 24082296 | 1.71E-05 |
|  |  | 16 | H3GA0046416 | 32300795 | 1.87E-05 |
|  |  | 16 | M1GA0020982 | 33082770 | 1.87E-05 |
|  |  | 16 | M1GA0020980 | 33106515 | 1.87E-05 |
|  |  | 16 | ALGA0090150 | 33139580 | 1.87E-05 |
|  |  | 17 | ALGA0095659 | 50745711 | 1.03E-05 |
|  |  | 17 | WU_10.2_17_57294356 | 50766364 | 1.03E-05 |
|  |  | 17 | WU_10.2_17_57113220 | 50907992 | 1.03E-05 |
| a* 24h | | 3 | WU_10.2_3_121580345 | 114315441 | 1.06E-05 |
|  |  | 3 | ALGA0020052 | 89860966 | 1.57E-05 |
| b* 45min | | 1 | ASGA0003137 | 59425153 | 3.63E-06 |
|  |  | 1 | ASGA0003191 | 62598591 | 8.16E-06 |
|  |  | 1 | ALGA0003901 | 62681226 | 8.16E-06 |
|  |  | 1 | MARC0065740 | 57399350 | 1.26E-05 |
|  |  | 1 | MARC0029325 | 57606300 | 1.26E-05 |
|  |  | 1 | ASGA0003069 | 57800971 | 1.26E-05 |
|  |  | 1 | DRGA0000963 | 58106684 | 1.26E-05 |
|  |  | 1 | INRA0002659 | 62534115 | 1.38E-05 |
|  |  | 1 | ASGA0003080 | 58140706 | 1.79E-05 |
|  |  | 1 | ALGA0003725 | 58162732 | 1.79E-05 |
|  |  | 1 | ASGA0003081 | 58188574 | 1.79E-05 |
|  |  | 1 | ALGA0003728 | 58209802 | 1.79E-05 |
|  |  | 2 | ALGA0014455 | 92482457 | 4.71E-06 |
|  |  | 2 | ALGA0014442 | 92001580 | 9.99E-06 |
|  |  | 2 | MARC0009432 | 91171772 | 1.34E-05 |
|  |  | 2 | M1GA0003027 | 91358118 | 1.34E-05 |
|  |  | 2 | MARC0030386 | 90853126 | 1.45E-05 |
|  |  | 3 | H3GA0008779 | 12416105 | 6.03E-06 |
|  |  | 3 | WU_10.2_3_12078711 | 12428306 | 6.03E-06 |
|  |  | 3 | ALGA0017562 | 12145439 | 6.71E-06 |
|  |  | 7 | ALGA0102690 | 67278108 | 3.9E-06 |
|  |  | 7 | MARC0066364 | 66641322 | 4.86E-06 |
|  |  | 7 | ASGA0034465 | 70886131 | 1.08E-05 |
|  |  | 7 | INRA0026451 | 72737984 | 1.33E-05 |
|  |  | 7 | MARC0090516 | 73038838 | 1.33E-05 |
|  |  | 8 | WU_10.2_8_24107361 | 23179828 | 1.53E-05 |
|  |  | 13 | MARC0021264 | 117690858 | 5.27E-07 |
|  |  | 13 | MARC0004754 | 118014718 | 6.41E-06 |
|  |  | 13 | DIAS0000441 | 121686307 | 7.38E-06 |
|  |  | 13 | WU_10.2_13_128631576 | 119253893 | 1.06E-05 |
|  |  | 13 | WU_10.2_13_128766109 | 119403365 | 1.06E-05 |
|  |  | 13 | MARC0037938 | 121907759 | 1.4E-05 |
|  |  | 13 | CASI0004415 | 57384026 | 1.49E-05 |
|  |  | 13 | ASGA0094034 | 56653519 | 1.58E-05 |
|  |  | 15 | WU_10.2_15_88484722 | 79162678 | 1.01E-05 |
| b* 24h | | 2 | WU_10.2_2_8181349 | 8838326 | 4.14E-07 |
|  |  | 3 | MARC0054644 | 58729525 | 4.76E-06 |
|  |  | 3 | MARC0017363 | 58088945 | 8.17E-06 |
|  |  | 3 | ALGA0111328 | 59214367 | 9.88E-06 |
|  |  | 3 | UMB10000086 | 59417014 | 9.88E-06 |
|  |  | 3 | ASGA0014850 | 59556077 | 1.25E-05 |
|  |  | 3 | ASGA0014790 | 57521426 | 1.45E-05 |
|  |  | 3 | H3GA0009670 | 57840432 | 1.45E-05 |
|  | 3 | | MARC0027326 | 59589286 | 1.54E-05 |
|  | 3 | | ASGA0014820 | 57887961 | 1.56E-05 |
|  | 3 | | ASGA0014862 | 59504883 | 1.56E-05 |
|  | 3 | | ASGA0014869 | 59642194 | 1.56E-05 |
|  | 6 | | ALGA0035889 | 88344172 | 1.88E-05 |
|  | 9 | | ALGA0054538 | 108068410 | 2.28E-07 |
|  | 9 | | WU_10.2_9_125279524 | 113821123 | 5.78E-06 |
|  | 9 | | WU_10.2_9_125241304 | 113780735 | 9.18E-06 |
|  | 9 | | ALGA0120289 | 113259009 | 9.55E-06 |
|  | 9 | | MARC0104613 | 105997671 | 1.19E-05 |
|  | 9 | | CASI0007330 | 110642210 | 1.54E-05 |
|  | 9 | | ASGA0099553 | 107842064 | 1.67E-05 |

**Table S2.** Significant SNP sites overlapped in both *F*st and GWAS analysis.

| Parameters | CHR | SNP | BP | Fst | P |
| --- | --- | --- | --- | --- | --- |
| L* 45min | 11 | rs80814916 | 41476323 | 0.42 | 1.76E-06 |
|  | 11 | rs81309786 | 42337689 | 0.42 | 3.25E-06 |
|  | 11 | rs80860230 | 41831777 | 0.42 | 4.47E-06 |
|  | 11 | rs80792496 | 41884238 | 0.42 | 4.47E-06 |
|  | 11 | rs80842640 | 41962603 | 0.42 | 4.47E-06 |
|  | 11 | rs80933877 | 42016587 | 0.42 | 4.47E-06 |
|  | 11 | rs80813136 | 42054759 | 0.42 | 4.47E-06 |
|  | 11 | rs80962818 | 42138666 | 0.42 | 4.47E-06 |
|  | 11 | rs1107726192 | 42196800 | 0.42 | 4.47E-06 |
|  | 11 | rs81235043 | 42215359 | 0.42 | 4.47E-06 |
|  | 11 | rs327980636 | 41821272 | 0.42 | 5.44E-06 |
|  | 11 | rs80963072 | 41458769 | 0.40 | 5.87E-06 |
|  | 11 | rs694158067 | 41441041 | 0.40 | 6.38E-06 |
|  | 11 | rs80796449 | 42398152 | 0.40 | 9.03E-06 |
|  | 11 | rs80789656 | 41772581 | 0.40 | 1.24E-05 |
|  | 11 | rs81431077 | 42151978 | 0.40 | 1.24E-05 |
|  | 11 | rs344381741 | 42187347 | 0.40 | 1.24E-05 |
|  | 11 | rs80909004 | 43676387 | 0.39 | 1.32E-06 |
|  | 11 | rs339600816 | 42922606 | 0.36 | 4.02E-06 |
|  | 11 | rs80925526 | 41250991 | 0.35 | 2.30E-06 |
|  | 11 | rs344012918 | 44005756 | 0.34 | 6.48E-07 |
|  | 11 | rs81313213 | 40874619 | 0.30 | 2.36E-06 |
|  | 11 | rs80920486 | 40995311 | 0.30 | 2.36E-06 |
|  | 11 | rs80847364 | 41014426 | 0.30 | 2.36E-06 |
|  | 11 | rs80815668 | 41097675 | 0.30 | 2.36E-06 |
|  | 11 | rs1107825159 | 41328254 | 0.30 | 2.36E-06 |
|  | 11 | rs1108758115 | 41361792 | 0.30 | 2.36E-06 |
|  | 11 | rs81295187 | 40134152 | 0.28 | 5.24E-07 |
|  | 11 | rs80948648 | 41123461 | 0.27 | 4.93E-06 |
|  | 11 | rs329985662 | 40691397 | 0.26 | 7.97E-07 |
|  | 11 | rs80825406 | 44662760 | 0.26 | 1.29E-05 |
|  | 11 | rs333100871 | 44556329 | 0.25 | 2.71E-07 |
|  | 11 | rs340052801 | 42772353 | 0.25 | 8.80E-07 |
|  | 11 | rs343148339 | 45309679 | 0.25 | 6.92E-06 |
| L* 24h | 3 | rs318541365 | 113665225 | 0.20 | 6.55E-06 |
| a* 45min | 2 | rs81360814 | 88083522 | 0.24 | 1.09E-05 |
|  | 2 | rs81360833 | 88166097 | 0.24 | 1.21E-05 |
| b* 45min | 2 | rs81361386 | 92482457 | 0.35 | 4.71E-06 |
|  | 2 | rs81361345 | 92001580 | 0.27 | 9.99E-06 |
|  | 2 | rs81245629 | 91171772 | 0.27 | 1.34E-05 |
|  | 2 | rs81361290 | 91358118 | 0.27 | 1.34E-05 |
|  | 2 | rs81225145 | 90853126 | 0.25 | 1.45E-05 |
|  | 13 | rs81289866 | 117690858 | 0.33 | 5.27E-07 |
|  | 13 | rs81235015 | 118014718 | 0.28 | 6.41E-06 |
|  | 13 | rs80943746 | 121907759 | 0.28 | 1.40E-05 |
|  | 13 | rs335398546 | 121686307 | 0.22 | 7.38E-06 |
|  | 15 | rs345536709 | 79162678 | 0.26 | 1.01E-05 |
| b* 24h | 6 | rs81389515 | 88344172 | 0.31 | 1.88E-05 |
|  | 9 | rs81281219 | 105997671 | 0.22 | 1.19E-05 |

**Table S3 Genes annotated by BioMart**

| Parameters | CHR | genes |
| --- | --- | --- |
| L* 45min | 11 | *PCDH9, KLHL1, DACH1, MZT1, BORA, DIS3, PIBF1, SNORA70, KLF5, KLF12* |
| L* 24h | 3 | *SELENOI, ADGRF3, HADHB, HADHA, GAREM2, RAB10, KIF3C, ASXL2, DNMT3A, EFR3B, DNAJC27, ADCY3, NCOA1, ITSN2, PFN4, TP53I3, SF3B6, FKBP1B, WDCP, MFSD2B* |
| a* 45min | 2 | *AP3B1, SCAMP1, LHFPL2, ARSB, DMGDH, BHMT, JMY, HOMER1, TENT2, CMYA5, MTX3, THBS4, SERINC5, U1, SPZ1, ZFYVE16, FAM151B, ANKRD34B* |
| b* 45min | 2 | *ACOT12, SSBP2, ATG10, RPS23, U6, SCARNA18, VCAN, HAPLN1, EDIL3* |
|  | 13 | *ZMAT3, U7, PIK3CA, KCNMB3, ZNF639, MFN1, GNB4, ACTL6A, MRPL47, NDUFB5, USP13, PEX5L, TTC14, CCDC39, FXR1, DNAJC19, ATP11B, MCCC1, MCF2L2, B3GNT5, SNORA63, KLHL24, YEATS2, MAP6D1, ABCC5, EIF2B5, DVL3, AP2M1, ABCF3, VWA5B2, ALG3, EEF1AKMT4, PSMD2, EIF4G1, SNORD66, FAM131A, CLCN2, POLR2H, THPO, CHRD, EPHB3, MAGEF1, VPS8* |
|  | 15 | *METAP1D, DLX1, DLX2, ITGA6, PDK1, RAPGEF4, MAP3K20, CDCA7, SP3, OLA1* |
| b* 24h | 6 | *MATN1, LAPTM5, SDC3, PUM1, NKAIN1, SNRNP40, ZCCHC17, FABP3, SERINC2, TINAGL1, HCRTR1, PEF1, COL16A1, ADGRB2, SPOCD1, PTP4A2, KHDRBS1, TMEM39B, KPNA6, TXLNA, CCDC28B, IQCC, DCDC2B, EIF3I, FAM167B, LCK*  *HDAC1, MARCKSL1, BSDC1, TSSK3, ZBTB8A, ZBTB8OS, SYNC, KIAA1522, YARS1, S100PBP, FNDC5, HPCA, TMEM54, RNF19B* |
|  | 9 | *KMT2E, SRPK2, PUS7, RINT1, ATXN7L1, CDHR3, SYPL1, NAMPT, CCDC71L, PIK3CG, PRKAR2B, HBP1, COG5* |

**Table S4. The type of SNPs identified near the candidate genes**

| Gene Name | CHR | Gene Position(Mb) | SNP | SNP Position(Mb) | Distance(Kb) | SNP Type |
| --- | --- | --- | --- | --- | --- | --- |
| *HOMER1* | 2 | 88.12-88.25 | rs81360814 | 88.08 | 39.35 | intron variant |
|  |  |  | rs81360833 | 88.16 | within | intron variant |
| *PIK3CG* | 9 | 106.69-106.74 | rs81281219 | 105.99 | 700.53 | intergenic variant |
| *PIK3CA* | 13 | 117.18-117.28 | rs81289866 | 117.69 | 413.62 | intergenic variant |
|  |  |  | rs81235015 | 118.01 | 737.48 | intron variant |
| *VCAN* | 2 | 91.35-91.81 | rs81361386 | 92.48 | 671.24 | intron variant |
|  |  |  | rs81361345 | 92.00 | 190.37 | intergenic variant |
|  |  |  | rs81245629 | 91.17 | 186.62 | intergenic variant |
|  |  |  | rs81361290 | 91.35 | 0.28 | non-coding transcript exon variant |
|  |  |  | rs81225145 | 90.85 | 505.27 | intergenic variant |
| *FABP3* | 6 | 87.94-87.95 | rs81389515 | 88.34 | 392.56 | intron variant |
| *FKBP1B* | 3 | 114.62-114.63 | rs318541365 | 113.66 | 958.30 | intron variant |


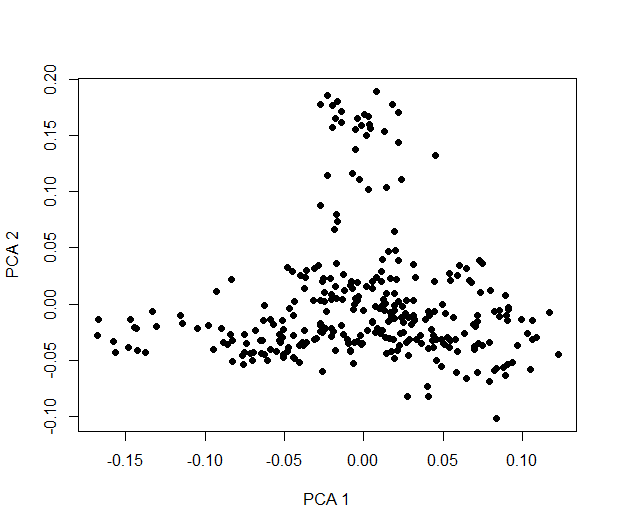


**Figure S1. PCA analysis of experimental population**
